# An acute bout of aerobic exercise reduces movement time in a Fitts' task

**DOI:** 10.1371/journal.pone.0210195

**Published:** 2018-12-31

**Authors:** Dean L. Smith, Randal P. Claytor

**Affiliations:** 1 Department of Kinesiology and Health, Miami University, Oxford, Ohio, United States of America; 2 Essence of Wellness Chiropractic Center, Eaton, Ohio, United States of America; Curtin University Faculty of Health Sciences, AUSTRALIA

## Abstract

Movement time (MT) is one of the most important variables influencing the way we control our movements. A few previous studies have generally found that MT reduces with reaction time testing during exercise. However, limited evidence exists concerning change in MT following an acute bout of exercise. Our purpose was to investigate the effect of an acute bout of aerobic exercise on movement time as assessed by a Fitts’ Law task. We also sought to determine if exercise would further lower MT during the more difficult task conditions compared with rest. Nineteen (12 male, 7 female) volunteers (19–28 yrs) completed a computerized paired serial pointing task to measure movement time before and after rest (R) and an acute bout of moderate aerobic exercise (E) using a within subjects crossover design. Comparisons between exercise and rest conditions were made to determine if there were differences in movement time. Exercise significantly reduced MT compared with rest. Movement time was reduced by an average of 208 ms following exercise compared with 108 ms following rest. Exercise did not further lower MT during the more difficult task conditions. These results suggest that an acute bout of aerobic exercise reduces movement time which is an important component of motor control. Further studies are needed to determine the duration of the effect as well as the optimum duration and intensity of exercise.

## Introduction

Movement time (MT) is a fundamental variable that reflects how movements are completed. MT is an inherent characteristic of motor control, but the principles underlying its formation (neural or computational) are in practice, poorly understood [1]. Slower movements could be costly to motor control for several reasons including delays in task achievement, reward acquisition and the potential monopolization of a significant amount of neural and attentional resources [1]. The brain’s energy supply determines its information processing power and the processing of sensorimotor input by the CNS comes at a metabolic expense [2]. From a practical perspective, the attentional cost associated with slow-paced movement could also interfere with the ability to achieve other tasks [2, 3].

Previous studies have generally found that MT is reduced with reaction time testing during exercise [4–7]. Studies that involve measures of response time (where response time equals reaction time plus movement time) tend to focus on either the reaction time component or the movement time component. In typical reaction time studies, the MT component of response time tends to be trivial such as with the press of a button where the MT component maybe less than 100 ms. The current study specifically investigated movement time *following* exercise as opposed to *during* exercise. The MT measured herein lasted anywhere between 1.5 to 2.5 seconds making these movements greater in duration compared with typical reaction time studies. While evidence is scarce concerning the effect of an acute bout of exercise on subsequent MT, there are many factors that may underlie the potential benefit of acute exercise on motor performance. For instance, studies show that physical exercise promotes changes in the human brain due to increases in metabolism [8], oxygenation and blood flow [9]. From a psychological viewpoint, exercise leads to increased arousal and cognitive resource allocation, resulting in improved performance on cognitive tasks [10]. Meta-analyses and narrative reviews offer support for the hypothesis that acute, moderate intensity exercise has a positive effect on speed of cognitive functioning [11]. More recently, motor learning in the form of improved movement accuracy [12] has been demonstrated following acute aerobic exercise. Additionally, exercise is known to result in changes of circulating levels of neuromodulators such as epinephrine, dopamine, serotonin, brain-derived neurotrophic factor (BDNF), and cortisol which may be important mediators of motor performance alteration [12, 13].

MT is defined as the time interval from the initiation of the response (e.g., mouse click) to the completion of the movement (e.g., subsequent mouse click). A frequent method of examining MT in the literature is by way of Fitts’ Law—a mathematical relation describing the speed-accuracy trade-off in motor skill performance. Fitts’ Law relates movement time to movement accuracy and distance and the relationship applies to many kinds of tasks [14] including discrete aiming movements, moving objects to insert them into a hole, moving a cursor on a computer screen [15–17], small finger movements under a microscope, and even throwing darts. A recent review article about the models of Fitts' Law notes that in all the areas of research into perceptual-motor control (motor cortex–plasticity), the work of Fitts (1954) has been by far the most influential and has initiated many hundreds of research papers [18]. Fitts’ Law has been used extensively in the field of ergonomics/human factors, and more recently in clinical research for example involving lumbar spinal stenosis patients and chiropractic patients [16, 19].

**Fig 1:**
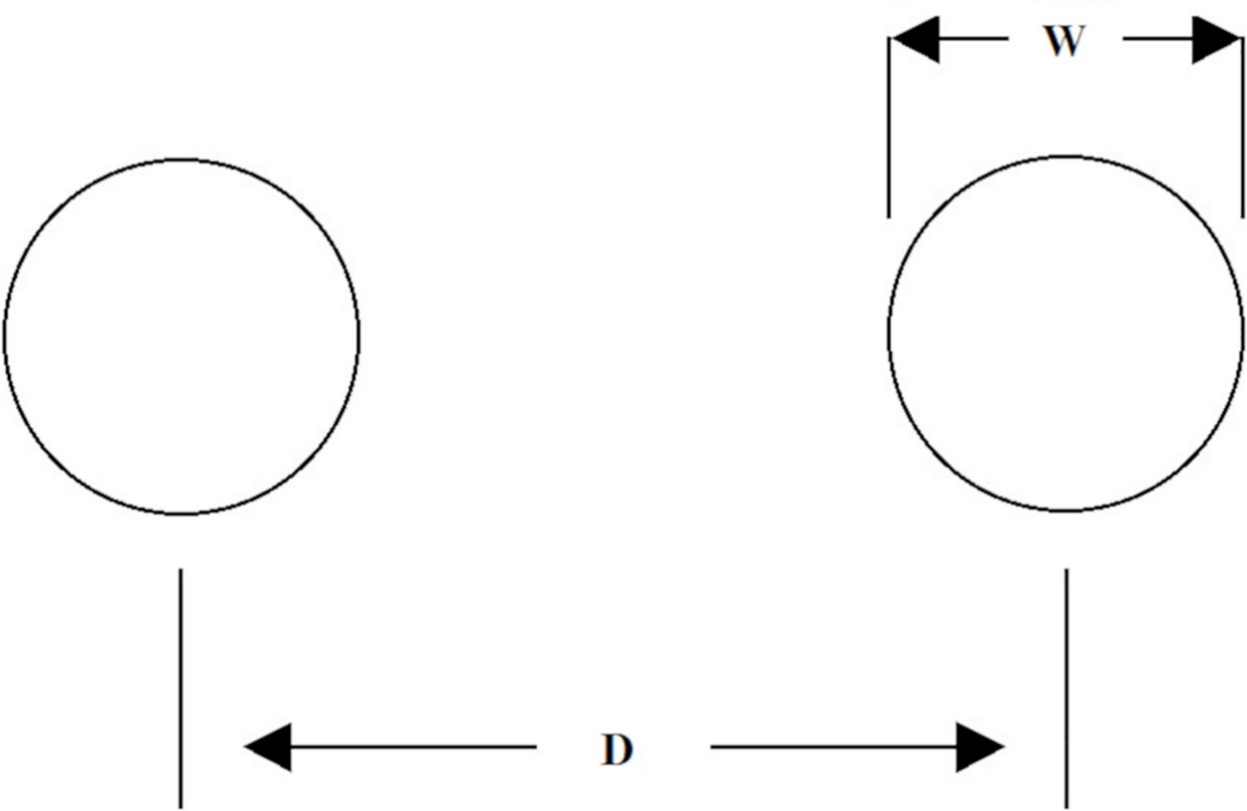
The Fitts’ task. The participant moves a cursor between two targets of width *W* separated by a distance *D*.

Fitts’ Law states that as target width (W) decreases or as distance (D) between targets increases (Fig 1), the movement time (MT) needed to acquire that target will increase in a linear fashion. The term Log_2_ (2D/W) has been called the Index of Difficulty (ID). Empirically, the relationship between MT and ID is linear with intercept a and slope b. Targets with low ID are “easier” since either the distance is less or the target is wider. Fitts’ Law can be expressed by the following equation: MT = a + b [Log_2_ (2D/W)]. With Fitts' Law having such a large volume of empirical investigation, its use by researchers who wish to investigate how exercise influences motor control is understood.

The aims of this study were to: 1) determine the effect of an acute bout of aerobic exercise on movement time. We hypothesized that an acute, moderate intensity, aerobic exercise intervention would reduce movement time compared with a rest condition; 2) determine if an acute bout of exercise has an influence on movement time as a function of level of task difficulty. We hypothesized that exercise would reduce MT during the more difficult task conditions (e.g., higher task difficulty) compared with rest.

## Materials and methods

### Participants

Nineteen (12 male, 7 female) college students between the ages of 19–28 (21.3 ± 2.0) years participated in this study. Given that there was no data available to conduct a standard sample size calculation in our population and given the within-subjects design, the sample size was estimated based on a similar study in a clinical population [16] as well as a study investigating head kinematics using the Fitts' paradigm [20]. The study protocol, all forms used and the informed consent documents were approved by the Human Subjects Institutional Review Board at Miami University. All participants read and voluntarily signed a written informed consent document and completed a health history questionnaire. All potential subjects were screened for inclusionary and exclusionary criteria prior to participation in the study. To be included in the study, participants were to be between the age of 18 to 30 years. Both aerobically trained and untrained participants were permitted into the study. Exclusion criteria included: a) those who exhibit two or more coronary heart disease (CHD) risk factors on the health history questionnaire; b) use of any medications (e.g. insulin, bronchodilators) that could affect physical performance or metabolism; c) any metabolic disease that would affect energy metabolism (e.g. diabetes); d) current smoker; e) any current orthopedic problems that would inhibit participation in experimental procedures. In addition to these criteria, participants were to abstain from caffeine and exercise for 3 hours prior to testing. Table 1 provides physical characteristics of study participants.

**Table 1:** Physical characteristics of the study participants.

	Mean	SD
**Age**	21.26	2.02
**Height (cm)**	171.61	8.13
**Mass (kg)**	70.82	16.42
**BMI**	23.87	4.82
**% Fat**	16.72	8.75
**Fat Mass (kg)**	12.01	8.02
**Lean Mass (kg)**	58.79	13.53
**VO2max (ml/kg/min)**	52.82	14.2

SD, standard deviation

### Design and procedure

**Fig 2:**
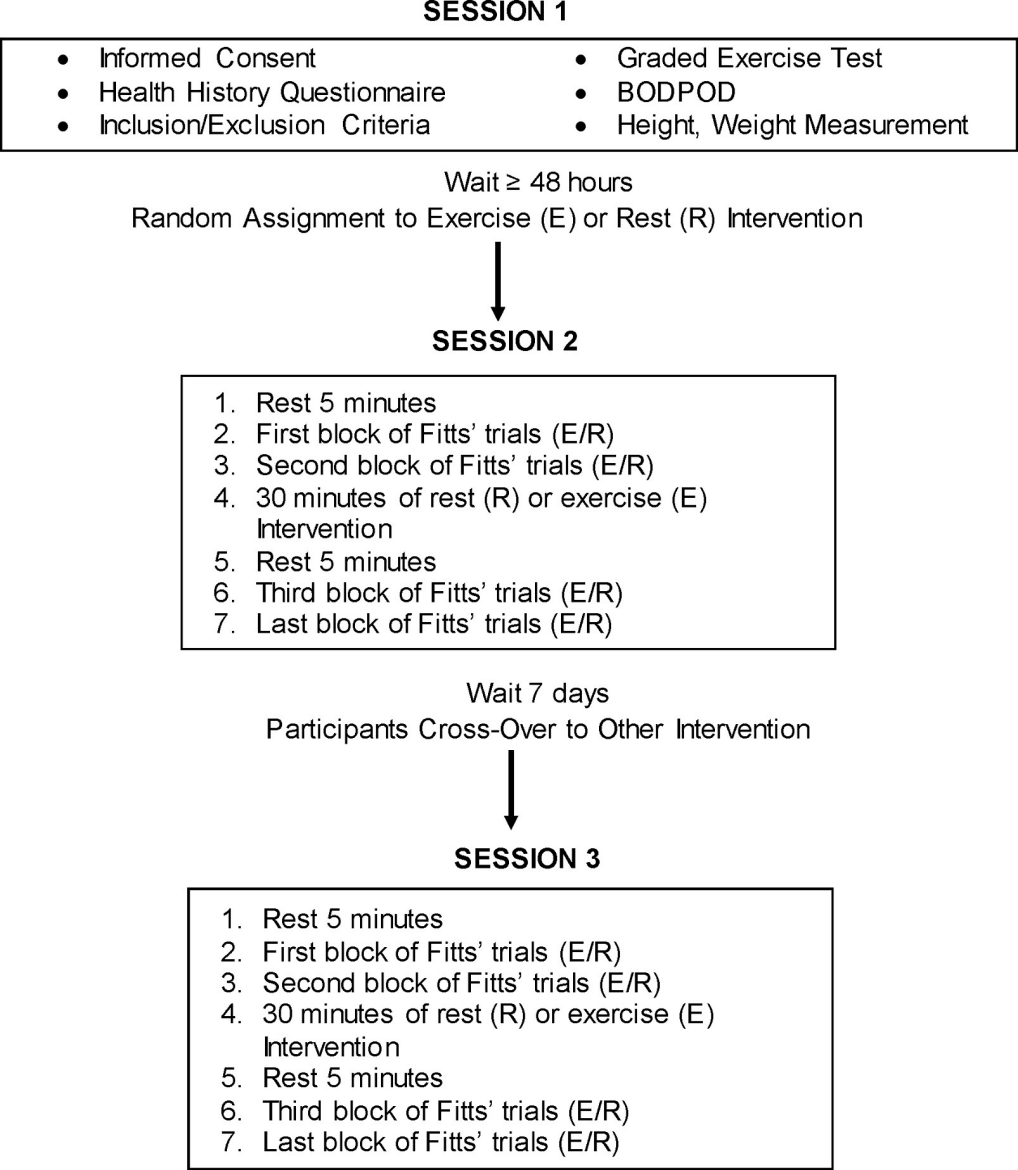
Flowchart of experiment timeline.

A flowchart of the experimental timeline is seen in Fig 2. For Session 1 –baseline testing, each participant underwent a graded exercise test on a treadmill to determine maximal aerobic capacity (VO2max), measurement of height and weight, assessment of body composition using a BODPOD and completed a physical activity questionnaire. Following Session 1, participants completed Fitts’ task testing before and after two intervention sessions—rest (R) and exercise (E) using a randomized, controlled, crossover experimental design. Session 2 was completed at least 48 hours after Session 1. Session 2 involved either the rest (R) intervention or exercise (E) intervention depending on the participant’s random assignment to intervention sequence. Time between Session 2 and Session 3 was approximately seven days.

Sessions 2 and 3 followed the same protocol. At the beginning of Sessions 2 and 3, participants completed 5 minutes of seated rest. Following the initial rest, participants completed two blocks each of 40 Fitts’ trials. Participants then either completed 30 minutes of rest (R) or 30 minutes of exercise (E) respectively. Five minutes after each intervention, participants repeated the two blocks of the Fitts’ task testing again.

### Baseline testing

Assessment of Body Composition, Height and Weight: Body fat percentage and the amount of lean body mass was obtained using air displacement plethysmography (BOD POD). Height was measured on a portable stadiometer (without shoes). Body weight was measured using an electronic platform scale while the subject was wearing only shorts and a t-shirt. Cardiovascular fitness was measured while performing exercise during a maximal graded treadmill test. The test began at a workload (3.5 mph and 0% grade) that the subject was easily able to perform and then increased by 1% grade every minute until the subject reached volitional fatigue or requested to stop. The graded exercise test was used to determine maximal oxygen consumption (VO2max) which was used to calculate the appropriate exercise level for the submaximal aerobic exercise intervention.

### Fitts’ Law task

**Fig 3:**
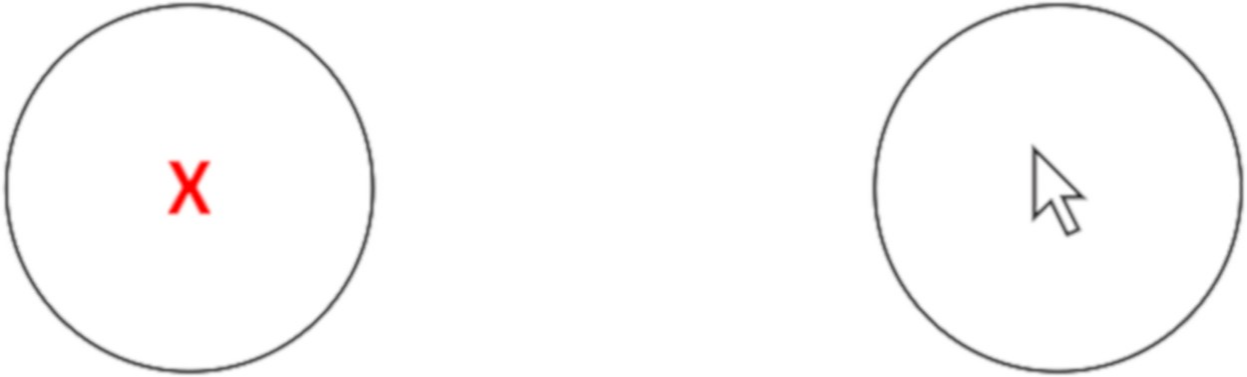
The Fitts’ task used in this experiment. A mouse click on a blank screen began timing for each trial at which time two circles appeared. A red X appeared in 1 circle designating it the target. The participant moved to this circle and clicked within it and then as quickly as possible moved back to the previous circle (which now was the target) and clicked within it.

A custom written program modeled after the Generalized Fitts’ Law Model Builder [16, 17, 21] was used to create a paired serial pointing task to measure movement time in milliseconds (Paradigm Stimulus Presentation, Perception Research Systems) [22]. Participants were seated in front of a computer and were instructed to use a mouse to move a cursor onto a circular target. The data were collected using a personal computer while sitting at arm’s length distance from the monitor. Each trial consisted of hitting a pair of circular targets as fast as possible. Participants were given the instructions of ‘move as quickly and accurately as possible’. A mouse click on a blank screen began timing for each trial at which time two circles appeared. A red X appeared in 1 circle designating it the target (Fig 3). The participant moved to this circle and clicked within it and then as quickly as possible moved back to the previous circle (which now was the target) and clicked within it. Timing then stopped, the screen went blank, and that pair was completed. This process continued until all 40 trials of that block were completed. The measured outcome from this task was the MT in milliseconds required to complete each of the trials. Circular targets were presented in pseudo-random order at 5 angles with respect to horizontal [16, 21, 23, 24]: 0, 45, 90, 135 and 180 degrees. All trials were conducted using a fixed distance (567 pixels) between targets. The program was configured to provide the serial pointing task with 4 width conditions (13, 27, 53, 106 pixels) that created four indexes of difficulty (3.4, 4.4, 5.4 and 6.5).

### Interventions

Exercise (E) consisted of walking or jogging at 65% of each participant’s maximal aerobic capacity for 30 minutes. Each subject’s workrate (i.e., treadmill speed and grade) was calculated based on 65% of VO_2_-Reserve and 65% of heart rate (HR) reserve (HR-Reserve). The first 3 minutes of exercise served as a progressive warm-up period to allow each subject to reach the workrate (treadmill speed and grade) that was calculated to elicit the desired cardiovascular and metabolic intensity. HR was monitored continuously throughout the exercise condition and VO_2_ was measured at minutes 4–8, 14–18 and 24–28. The workrate (i.e., speed and/or grade) was adjusted, if necessary, to maintain the same relative intensity (i.e., 65% of capacity) throughout the exercise condition. Rest (R) consisted of reading or listening to music for 30 minutes in a semi-reclined position. HR was monitored continuously throughout the rest condition. As with any motor task, some improvement was expected because of practice. The resting control intervention made possible the testing for MT reduction as a result of the passage of time and practice.

We chose to use walking as the mode of exercise in this study because it is a ubiquitous form of physical activity and because we could control the intensity of the activity precisely by manipulating the speed and the grade of walking so that the relative intensity of exercise was the same for each subject. Cognitive and motor performance may be differentially affected by exercise mode. For instance, cycling has been shown to be associated with improved performance during and after exercise, whereas treadmill running impaired performance during exercise and led to a small improvement in performance following exercise [10].

### Data analysis

Statistical analyses were performed using paired t-tests and a one-way, repeated measures ANOVA at each index of difficulty level. All analyses were conducted using IBM SPSS Statistics for Windows, Version 21.0. Statistical significance was accepted at p < 0.05. Possible outliers in the data, were assessed by inspection of a boxplot (for values greater than 1.5 box-lengths from the edge of the box). Normality was assessed by Shapiro-Wilk’s test and an exact sign test was used if normality was violated.

## Results

### Testing baseline values

We performed a paired t-test using the first block of 40 trials (pre) to determine if there were baseline differences between R and E. There were no outliers in the data, as assessed by inspection of a boxplot (for values greater than 1.5 box-lengths from the edge of the box). The differences between the first block of trials in the exercise session and the first block of trials in the rest session were not normally distributed, as assessed by Shapiro-Wilk's test (p = .027). As a result, we ran an exact sign test to compare the baseline differences between E and R. We found that there was no statistically significant median difference between baseline E and R values, exact p = 1.0, indicating no difference between rest and exercise conditions.

### Testing whether exercise would reduce movement time compared with rest

**Fig 4:**
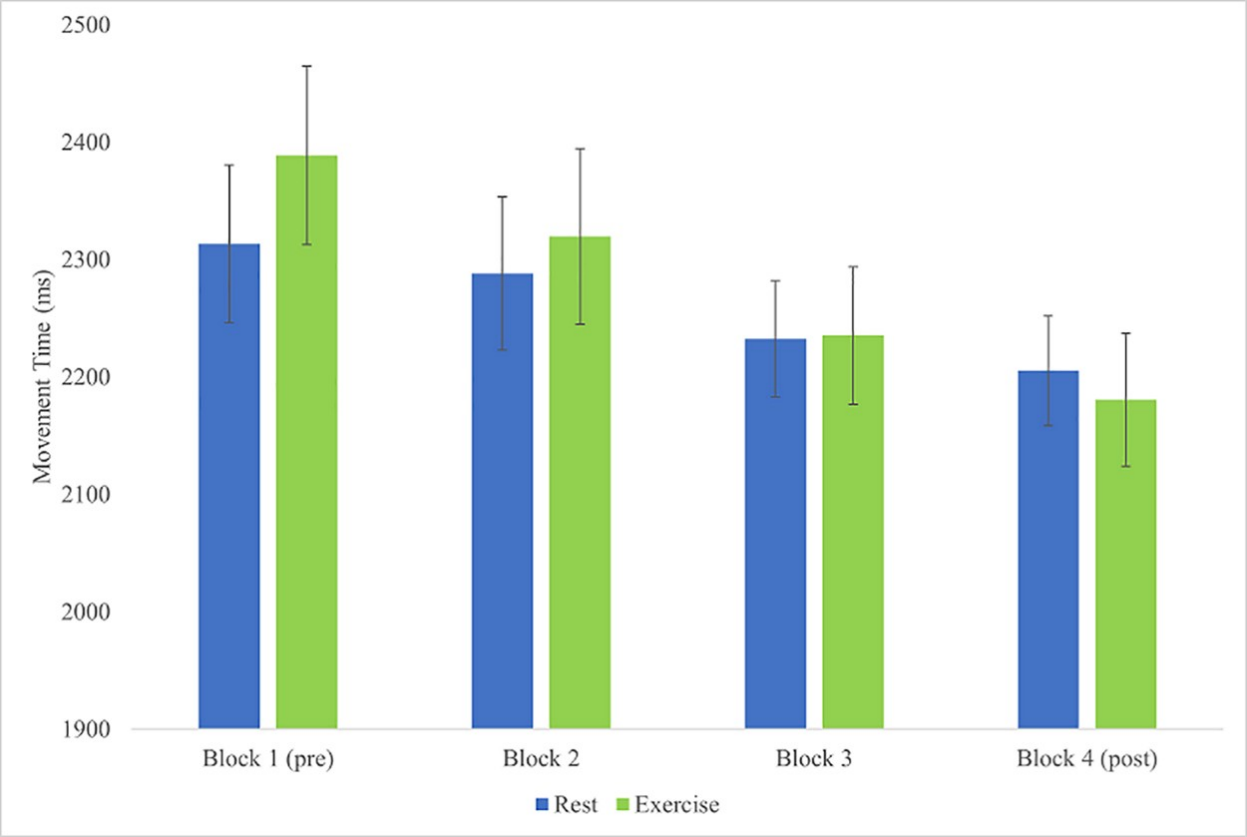
Differences in MT by intervention and block (mean ± SE).

We conducted a paired t-test to compare the delta (change) scores between the difference of the *first block (pre) and last block of trials* (post) for exercise to the difference of the *first block (pre) and last block* of trials (post) for rest. Fig 4 shows the differences in MT by intervention and block.

Specifically, we computed the difference scores for exercise (pre-post) and rest (pre-post) and entered these into the analysis. There were no outliers in the data, as assessed by inspection of a boxplot (for values greater than 1.5 box-lengths from the edge of the box). The change scores between the two interventions were normally distributed, as assessed by Shapiro-Wilk's test (p = .529). Reductions in MT occurred for both the exercise condition (208.46 ± 127.88 ms) and rest condition (108.04 ± 172.56 ms). A statistically significant reduction in MT of the exercise intervention compared with the rest intervention of 100.42 (95% CI, -173.98 to -26.86) ms, t(18) = -2.868, p = .010, d = 0.658. This equates to a 4.67% reduction in MT after rest, compared with an 8.73% reduction in MT with exercise.

### Testing if exercise would reduce MT during the more difficult task conditions compared with rest

A one-way repeated measures ANOVA was computed to determine if exercise would lower MT during the more difficult task conditions (e.g., higher ID) compared with rest. The dependent measure used in this analysis was the MT delta score between pre-post exercise and pre-post rest as measured by the following equation: Δ MT (ms) = (Epost-Epre)—(Rpost-Rpre). The within-subjects factor was ID which had four levels (3.4, 4.4, 5.4 and 6.5). MT delta scores were normally distributed at each index of difficulty level as assessed by Shapiro-Wilk's test (p > .05). Mauchly's Test of Sphericity indicated that the assumption of sphericity had been violated, and therefore, Greenhouse-Geisser corrections were used. There was no significant effect of ID, F(1.56, 28.13) = 0.84, p = .42, η2p = .04, such that exercise did not further lower MT at differing levels of ID compared with rest.

## Discussion

Despite the potential clinical and practical relevance (e.g., faster movements, more time for cognitive decision making, greater efficiency), relatively few studies have examined changes in motor behavior following a single session of exercise. We sought to determine the effect of an acute bout of aerobic exercise on MT. In this study MT was significantly reduced immediately following 30 minutes of moderate aerobic exercise as compared with 30 minutes of rest. As predicted by Fitts’ Law, when the difficulty of the task increased, movement time for both rest and exercise also increased. However, there was no differential effect of the index of difficulty on MT with exercise. Therefore, the results indicate that exercise reduced movement time regardless of the task difficulty. Specifically, there was a 4.7% reduction in MT after rest, compared with an 8.7% reduction in MT following an acute bout of exercise. These results suggest that an acute bout of aerobic exercise influences an important component of motor control, although further studies are required to determine the duration of this effect on MT.

One other study used a Fitts' specific task to quantify MT following any kind of physical activity. A study by Passmore et al [19] examined Fitts’ task performance before and after, four speeds of walking over a 12-minute interval in healthy and lumbar spinal stenosis (LSS) patients. Results of the study [19] indicated that walking improved reaction time on the task for both groups and that MT was significantly slower in those with LSS. LSS participants exhibited longer MTs than healthy participants, an effect that was amplified at larger IDs, but no main effect of exercise was found. Another study [12] used a Fitts’ like method to examine the speed-accuracy tradeoff following 30 minutes of moderate intensity running on a Sequential Visual Isometric Pinch Task (SVIPT). The authors of this study found an immediate improvement in motor acquisition for both a single session and on multiple sessions across subsequent days. However, that study found that the effect was driven by improved movement accuracy, as opposed to speed which contrasts to the results of our study where movement time was reduced. The difference between this study and ours might be explained by the methods used—pinch force to control cursor movement [12] vs. hand (mouse) movements to control cursor movement. The Statton et al. [12] study used a novel, isometric pinch task that is sufficiently difficult to ensure that performance continues to improve over 5 days of training [25]. While Fitts' Law could also be extended to the realm of isometric force control [26], this contrasts to our use of a commonplace, isotonic/dynamic task. The tasks also differ in that limb mass plays a role in moving the mouse (cursor) with that movement affecting the afferent information available during the unfolding of the action [26]. Such afferent information from dynamic movement would be unavailable in the pinch task.

While the neural mechanisms driving the cortical changes postexercise are still unclear [27], it seems possible that intensity is a key modifier of the effects of acute aerobic exercise on changes in complex motor behavior [28]. For example, the work by Roig et al. [29] stresses the importance of high intensity exercise for motor learning, whereas an acute bout of moderate-intensity aerobic exercise may facilitate the maintenance of motor performance during skill acquisition [28] as well as the motor preparatory components of movement [27]. Further work is necessary to establish a dose-response relationship and intensity-based relationship between aerobic exercise and motor control [28].

Recent evidence shows that exercise influences the excitability of neurons in the primary motor cortex [30]. Transcranial magnetic stimulation (TMS) studies suggest that exercise suppresses inhibitory and promotes excitatory intracortical networks that impact corticospinal tract output [31]. With aerobic exercise, modulation of these intracortical networks may augment corticospinal tract output leading to heightened electromyographic activity and a decrease in movement time [31]. Acute aerobic exercise appears to modulate primary motor cortex inhibition and it is becoming increasingly evident that GABA-mediated inhibition is transiently reduced post-exercise [32], but whether this mechanism can explain changes in MT is unclear.

Studies using electroencephalography (EEG) have found changes in the event related potentials (P3 component) immediately following aerobic exercise during a variety of task performances [27, 33]. The P3 component is thought to reflect the synchronized firing of large groups of excitatory pyramidal neurons of the cortex [33]. It also appears to be modulated by interactions between the frontal and temporal/parietal cortices [34]. Collectively, EEG evidence suggests that one mechanism underlying the acute exercise-induced improvements in cognition may be the enhancement of synchronous firing between cortical neurons [33].

Acute, moderate intensity aerobic exercise such as that used in this study appears to have a positive effect on the speed of performance of a variety of cognitive tasks [35]. A single session of exercise has been shown to increase cortical excitability and improve performance on tasks of executive function [30]. Neurologically, the positive impact of acute exercise on the prefrontal and primary motor cortices [30] may explain the results obtained in this study. Previous neuroimaging evidence has shown that the regulation of speed-accuracy responses is associated with functioning of prefrontal regions [36] including association areas as well as the pre-supplementary motor area [37]. Cortical activity underlying Fitts’ Law has also been observed in monkeys implanted with multi-electrode arrays in the primary motor (M1) and primary somatosensory (S1) cortices [38]. The monkeys performed reaches with a joystick-controlled cursor toward different sized targets. The reaction time (RT), MT, and movement velocity changed with target size, and M1 and S1 activity reflected these changes [38]. Based upon existing evidence it seems reasonable that exercise reduced MT on a Fitts’ task in this study, at least partly due to its impact on prefontal and primary motor cortices and exercise likely acted as a priming mechanism to enhance motor function.

The positive effects of acute and long-term exercise on neural plasticity, learning, and memory, have led to recent investigations of aerobic exercise as a priming mechanism to enhance motor function [39]. In addition to the study using the Sequential Visual Isometric Pinch Task (SVIPT) [12], Roig et al. [29] demonstrated that 20 min of intense cycling completed immediately before or after performing a visuomotor skill task improves retention of the motor skill more than practicing the task alone. It has been suggested that when exercise comes before practice, improvements in skill learning might be due to better encoding of procedural information whereas when exercise is performed after practice the effect may be due to consolidation of long-term memory [39, 40].

Aerobic exercise is a cost-effective way to enhance the lives of humans. It is emerging that exercise also facilitates the capacity of the neuromuscular system to be more sensitive to task specific training as well as optimizing motor learning and recovery [39]. Our study along with those of Roig et al. [29] and Statton et al. [12] also suggest acute performance benefits of movement-based priming. The evidence to date provides justification as to why individuals may choose to incorporate acute bouts of exercise as part of their routine to improve sensorimotor performance on subsequent tasks. This study adds to the growing literature regarding how exercise affects movement performance, in particular movement time.

## Conclusion

An acute bout of moderate intensity, aerobic exercise led to a significant reduction in movement time (MT) compared with a resting control condition. The results obtained herein as well as the popularity of the Fitts’ Law paradigm in other disciplines argues for its continued investigation of the effect of exercise on human motor behavior. Exercise scientists and clinicians should further consider and assess how exercise may impact movement time–an important component of sport performance and daily activities. Further research examining the duration of movement time reduction following an acute bout of exercise is needed in addition to the optimum duration and intensity of exercise for movement-based priming.

## Supporting information

S1 Dataset Demographics and movement time. (XLSX)
